# Has a fast treatment transition from surgical to endovascular operations improved the survival of aneurysmal subarachnoid hemorrhage?

**DOI:** 10.1007/s00701-025-06447-1

**Published:** 2025-02-04

**Authors:** Aleksanteri Asikainen, Ilari Rautalin, Rahul Raj, Miikka Korja, Mika Niemelä

**Affiliations:** 1 Department of Neurosurgery, University of Helsinki and Helsinki University Hospital, P.O. Box 320, Helsinki, FI-00029 Finland; 2 The National Institute for Stroke and Applied Neurosciences, Auckland University of Technology, Private Bag 92006, Auckland, 1142 New Zealand

**Keywords:** Subarachnoid hemorrhage, Case fatality, Endovascular treatment, Surgical treatment, Temporal trends

## Abstract

**Background:**

Several studies have attributed decreasing case fatality rates (CFRs) of aneurysmal subarachnoid hemorrhage (aSAH) to the gradually increasing use of endovascular treatment without considering improvements in other outcome-affecting factors. To assess the independent effect of a treatment modality on CFRs, we investigated CFR changes in a high-volume center rapidly transitioning from surgical to endovascular operations as the first-line treatment for all aSAH patients except those with middle cerebral artery (MCA) aneurysms.

**Methods:**

We identified all surgically/endovascularly treated aSAH patients in Helsinki University Hospital (HUH) during 2012–2017. As the treatment shift occurred in 2015, we defined two treatment eras: surgical (2012–2014) and endovascular (2015–2017). We compared time-dependent changes in 1-year CFRs between non-MCA and MCA patients using a Poisson regression model. To analyze consistency in operation rates, we also identified sudden-death and conservatively treated aSAHs in the HUH catchment area via two externally validated registers.

**Results:**

Of all 665 hospitalized aSAH cases in the HUH catchment area, 557 (84%) received operative treatment; 367 (66%) underwent surgical and 190 (34%) endovascular operations. Between the treatment eras, endovascular treatment for non-MCA cases increased from 21 to 79%, whereas 99% of the MCA cases were treated surgically during the whole study-period. Among the operatively treated patients, the 1-year CFRs decreased similarly in patients with non-MCA (42%; from 14 to 8%; adjusted risk ratio (aRR) = 0.66 (95% CI 0.37–1.19)) and MCA aneurysms (42%; from 15 to 9%; aRR = 0.66 (0.16–1.60)). The proportion of operatively treated patients, their clinical condition on admission, and amount of bleeding on the first CT-scan remained unchanged over time.

**Conclusions:**

We found similar CFR decreases in aSAH groups with and without undergoing a fast transition from surgery to endovascular operations, providing real-world evidence on the small independent effect of endovascular treatment on the decreasing CFRs in high-volume centers.

**Supplementary Information:**

The online version contains supplementary material available at 10.1007/s00701-025-06447-1.

## Introduction

Two decades ago, the International Subarachnoid Aneurysm Trial (ISAT) reported that the endovascular coiling of ruptured intracranial aneurysms (RIA) was associated with a more favorable outcome compared to neurosurgical clipping among aneurysmal subarachnoid hemorrhage (aSAH) patients suitable for both treatment modalities [25]. Consequently, numerous neurosurgical centers shifted their first-line treatment of RIAs towards endovascular operations [20, 21, 27, 30, 34], which has been considered one of the main reasons for the decrease in case fatality rates (CFRs) of aSAH in the 21st century [23, 26]. However, while most high-volume centers implemented this shift gradually over several years [20, 21, 34], other factors affecting the outcome of aSAH such as patient demographics, environmental risk factors, diagnostic modalities and nonoperative acute care have also changed simultaneously. Therefore, the real-world evidence for the independent effect of endovascular treatment on decreasing CFRs of aSAH has remained limited.

In contrast to many other ISAT-like centers that have expertise in surgical and endovascular management of RIAs and handle over 60 cases annually, the neurosurgical center of the Helsinki University Hospital (HUH) underwent a fast first-line treatment shift of RIAs from surgical to endovascular operations for all except middle cerebral artery (MCA) aneurysms [7], while simultaneously monitoring the rates of conservatively treated aSAH patients and aSAH cases dying before hospitalization in the same hospital catchment area. This unique real-world study setting allowed us to investigate whether the CFR trends between aSAH patients with MCA and non-MCA aneurysms differed substantially over the fast first-line treatment transition in HUH, while controlling for the changes in conservatively treated and sudden-death aSAH cases. We hypothesized that CFR changes were similar between these groups.

## Methods

### Ethical consideration

This retrospective study was approved by the HUH institutional review board, the Finnish Institute for Health and Welfare (THL/3136/14.06.00/2021), and Statistics Finland (TK/2920/07.03.00/2023). The requirement to obtain patient consent was waived by all three institutions due to the secondary use of already-collected health data. This study was performed in accordance with the Declaration of Helsinki.

### Study setting and population

The Department of Neurosurgery at HUH is a well-established, public, and nonprofit neurosurgical center responsible for the treatment of all aSAH patients in Southern Finland, encompassing a catchment area of approximately 2.2 million people. Based on an electronic health record search using procedure-specific codes, we identified all aSAH patients admitted to the HUH neurosurgical unit between January 2012 and December 2017, who underwent either microsurgical clipping or endovascular treatment for aneurysm repair. We excluded patients with previous aSAHs, previously treated unruptured aneurysms with a delayed rupture, an aneurysm treated primarily with bypass, and patients with arteriovenous malformations. At HUH, microsurgical clipping was the primary choice of treatment for all ruptured and unruptured aneurysms until 2015. After this period, the chairman of the department changed and the treatment policy was rapidly shifted to favor endovascular operations as the primary choice of treatment for all aneurysms, except those located in the MCA [7]. Thus, we defined two treatment eras: (1) the surgical era during 2012–2014, and (2) the endovascular era during 2015–2017. We further divided the aSAH patients into two groups by aneurysm location: (1) the MCA group, and (2) the non-MCA group. Although the choice to perform surgical or endovascular treatment was predominantly based on the period-specific policy, the final decision was made based on patient and aneurysm-specific characteristics in a multidisciplinary meeting.

### Sudden deaths and conservatively treated patients

To examine whether aneurysm repair rates were consistent between the two treatment eras, we also used two administrative nationwide registers to identify aSAH cases that did not undergo aneurysm repair. The details of the register-based aSAH case ascertainment and its external validation have been published elsewhere [2, 3, 29]. Briefly, we combined the data of the national Care Register for Health Care (CRHC) and the national Cause of Death Register (CDR) to identify the fatal and non-fatal aSAHs (using ICD-10 codes I60.0–I60.6) in the HUH catchment area. Hospitalized aSAH events in the CRHC have been validated with a positive predictive value (PPV) of 99.8% compared to a hospital-based aneurysm register [19]. The CDR has in turn been externally validated with a PPV of 97% for fatal SAHs [33]. In the current study, we first ascertained aSAH patients who died before reaching hospital wards (defined as sudden deaths, which account for over 50% of fatal aSAHs [2]) through the CDR. Using the CRHC, we then extracted data on all aSAH patients who were admitted/transferred to HUH neurosurgery or other hospital departments without aneurysm repair. Among the conservatively treated patients who were admitted to HUH neurosurgery, we identified organ donors through a prospectively collected database governed by the HUH transplantation office.

### Data collection and outcomes

For aSAH patients who underwent aneurysm repair, we gathered clinical data retrospectively through electronic health records. Specifically, we extracted data on age, sex, date of hospital admission, aneurysm location and size (maximum diameter), use of external ventricular drainage (EVD), presence of intracerebral hemorrhage (ICH), smoking status, history of hypertension, treatment modality, and multiplicity of treated aneurysms. Moreover, we categorized patients by aSAH severity into good-grade (World Federation of Neurological Surgeons (WFNS) grade 1–3) and poor-grade groups (WFNS grade 4–5) [9], while also distinguishing patients with thin/absent (modified Fisher scale 0–2) and thick bleeding (modified Fisher scale 3–4) on the first CT scan after hospital admission [10]. Definitions for the collected variables are provided in Supplementary Table 1. The date and place of death of fatal aSAHs were extracted from the CDR. The outcomes of interest were the all-cause 30-day and 1-year CFRs.

## Statistical analyses

To analyze differences in baseline characteristics between treatment eras, we used the Fisher exact test for categorical variables, whereas for continuous variables, we used the Mann-Whitney U test for nonparametric data. In terms of outcomes, we calculated 1-year (primary outcome measure) and 30-day CFRs (secondary outcome measure) by aneurysm location (MCA vs. non-MCA group) and treatment era (surgical era in 2012–2014 vs. endovascular era in 2015–2017). We used a Poisson regression model to calculate risk ratios (RRs) with 95% confidence intervals (CIs) for fatal aSAH among operatively treated patients between treatment eras and in the two aneurysm location groups. In addition to age and sex, we considered variables that associated with the CFRs and changed differently between the aneurysm location groups over the treatment transition as possible confounders; these were included in the adjusted multivariable models. Moreover, we used a likelihood ratio test to investigate whether the adjusted CFR trends differed between aneurysm groups. To examine time-dependent changes in patient selection, we calculated the proportions of sudden deaths, organ donors, patients admitted to HUH neurosurgery, patients who underwent aneurysm repair, as well as patients admitted to other treatment units, and compared these proportions by treatment era using the Fisher exact test. We used Stata v17.0 (StataCorp) for statistical analyses.

## Results

### Patient selection by treatment era

**Fig. 1:**
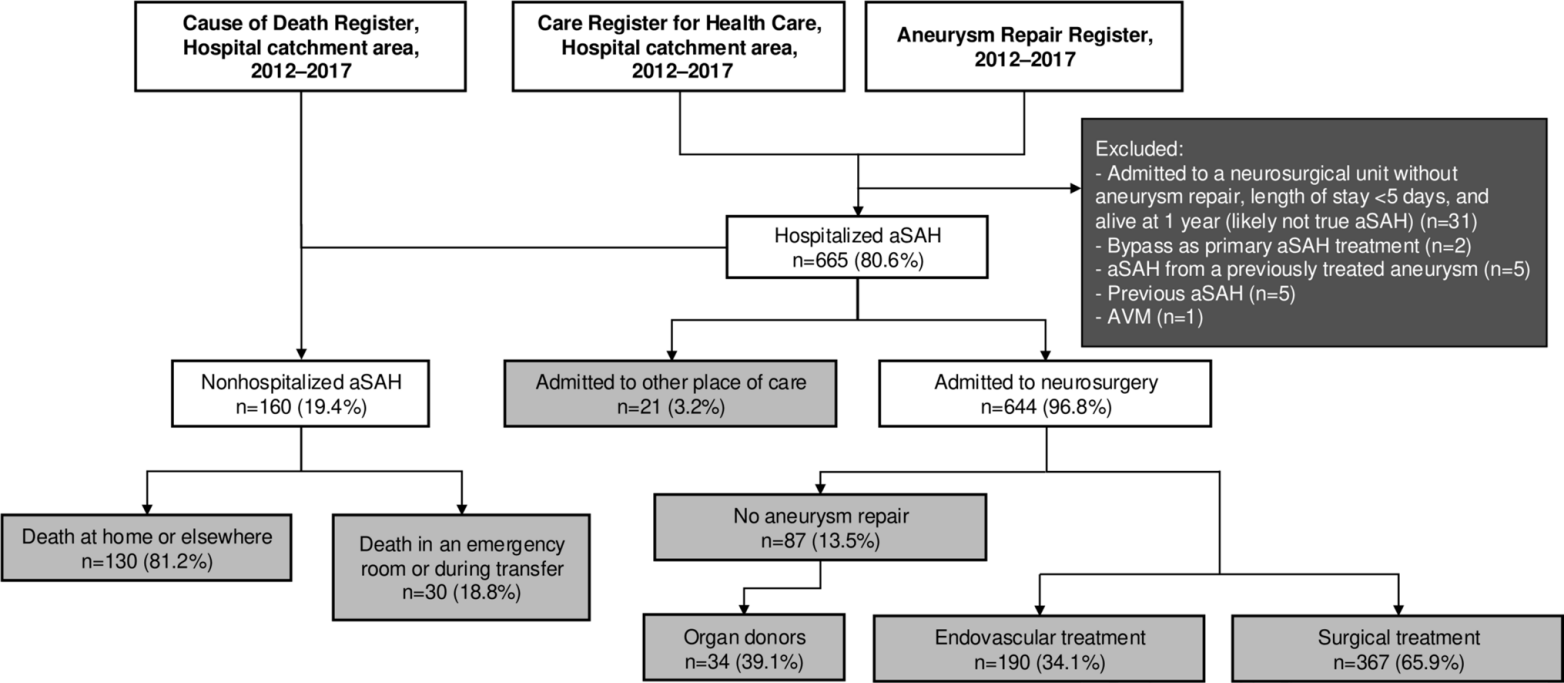
Flowchart of aSAH case identification strategy. Abbreviations: aSAH = aneurysmal subarachnoid hemorrhage; HUH = Helsinki University Hospital; AVM = arteriovenous malformation. The light gray boxes indicate aSAH cases included in the final cohort, and the dark gray box indicates aSAH cases excluded from the final cohort

**Fig. 2:**
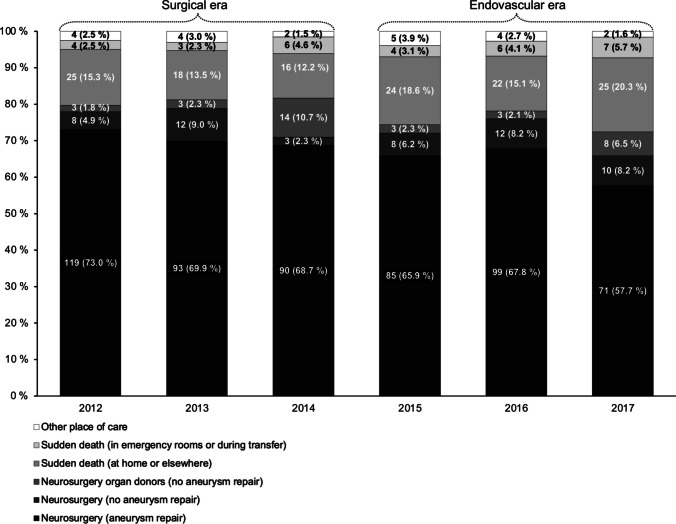
Overall distribution of aSAH patients’ place of care and/or death by treatment era. Abbreviations: aSAH = Aneurysmal subarachnoid hemorrhage

During 2012–2017, we identified a total of 825 first-ever aSAH cases in the catchment area of HUH, of which 130 (15.8%) died at home or elsewhere, and 30 (3.6%) died in emergency rooms or during transfer. Of the 665 hospitalized patients, 644 (96.8%) were admitted to HUH neurosurgery, of which 557 (86.5% of patients admitted to HUH neurosurgery) underwent repair operation for RIA (Fig. 1). Among the 695 patients who did not die at home or elsewhere, the proportion of aSAH cases who underwent aneurysm repair did not significantly change between the treatment eras (from 82.1% (95% CI 77.8–85.9%) to 78.0% (95% CI 73.1–82.4%), *p* = 0.184) (Fig. 2). Simultaneously, the proportion of organ donors among conservatively treated fatal cases in HUH neurosurgery decreased by 41.6% (from 80.0% (95% CI 59.3–93.2%) to 46.7% (95% CI 28.3–65.7%), *p* = 0.014).

## Patient characteristics by treatment era and location group

**Fig. 3:**
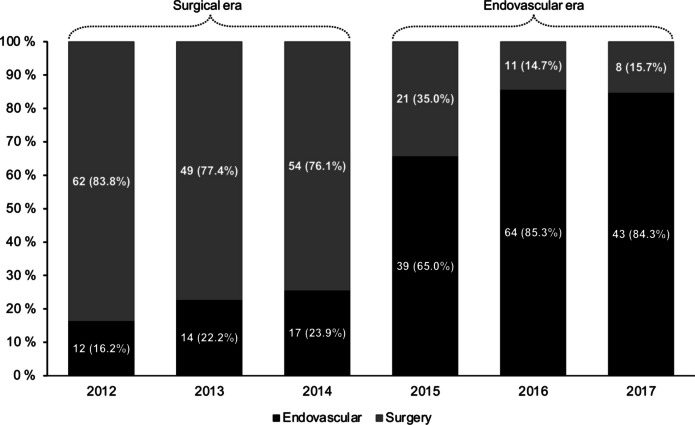
Distribution of operative treatment strategies for non-MCA aSAH cases by treatment era. Abbreviations: aSAH = Aneurysmal subarachnoid hemorrhage; MCA = Middle cerebral artery

Between 2012 and 2017, 367 (65.9%) aSAH patients underwent surgical clipping and 190 (34.1%) underwent endovascular treatment. In total, 302 (54.2%) patients were treated in the surgical era (2012–2014) and 255 (45.8%) in the endovascular era (2015–2017) (Table 1). In terms of aneurysm location of surgically and endovascularly treated patients, 94 (31.1%) had an MCA aneurysm in the surgical era and 69 (27.1%) in the endovascular era (Table 1). In comparison to non-MCA cases, patients with MCA aneurysms were younger (median 54.3 years vs. 57.7 years, *p* = 0.025), more commonly women (75.5% vs. 61.9%, *p* = 0.002), had larger aneurysms (median 9.0 mm vs. 6.0 mm, *p* < 0.001), and more commonly experienced ICHs (54.0% vs. 29.2%, *p* < 0.001). In addition, they underwent endovascular aneurysm repair less frequently (0.6% vs. 48.0%, < 0.001), had fewer insertions of EVD (28.2% vs. 44.4%, *p* < 0.001), and received operative treatment of only one aneurysm less often (80.4% vs. 92.9%, *p* < 0.001) (Table 1). Between the surgical and endovascular eras, we found significant temporal changes in the proportion of women in the non-MCA group (67.3% vs. 56.3%, *p* = 0.022), median aneurysm size in the MCA group (9.0 mm vs. 7.0 mm, *p* = 0.007), the use of endovascular treatment in the non-MCA group (20.7% vs. 78.5%, *p* < 0.001), and the proportion of non-MCA cases undergoing simultaneous treatment for multiple aneurysms (10.1% vs. 3.8%, *p* = 0.018) (Table 1) (Fig. 3). Otherwise, all patient characteristics remained similar over time (Table 1). All cases except one MCA (99.4%) were treated surgically during the whole study period, whereas the proportion of endovascularly treated posterior circulation aneurysms increased from 32.4% in the surgical era to 94.6% in the endovascular era (Table 1) (Supplementary Table 2).

**Table 1:** aSAH characteristics among operatively treated patients by treatment era and aneurysm location

	MCA cases			non-MCA cases			Difference between all MCA and non-MCA cases
	Surgical Era (2012–2014)	Endovascular Era (2015–2017)	*P*-value	Surgical Era (2012–2014)	Endovascular Era (2015–2017)	*P*-value	*P*-value
No. aSAHs	94	69		208	186		
Median age at treatment, year (IQR)	52.3 (46.0–62.9)	56.7 (48.9–66.0)	0.120	58.8 (50.2–64.9)	56.0 (49.0–65.3)	0.130	**0.025**
Women, *n* (%)	70 (74.5)	53 (76.8)	0.854	140 (67.3)	104 (55.9)	**0.022**	**0.002**
Aneurysm location, *n* (%)							
ACom/A1	N/A	N/A	N/A	93 (44.7)	98 (52.7)	0.325	N/A
Pericallosa	N/A	N/A		13 (6.3)	8 (4.3)		
ICA	N/A	N/A		65 (31.3)	43 (23.1)		
VBA/PCA	N/A	N/A		24 (11.5)	23 (12.4)		
PICA/AICA/SCA	N/A	N/A		13 (6.3)	14 (7.5)		
Aneurysm size (mm), Median (IQR)	9.0 (6.0–12.0)	7.0 (5.0–9.1)	**0.007**	5.6 (4.0–8.2)	6.0 (4.0–8.0)	0.749	**< 0.001**
WFNS grade, *n* (%)							
1–3	60 (63.8)	37 (53.6)	0.201	140 (67.3)	127 (68.3)	0.914	0.064
4–5	34 (36.2)	32 (46.4)		68 (32.7)	59 (31.7)		
Modified Fisher grade, *n* (%)							
0–2	22 (23.4)	12 (17.4)	0.436	39 (18.8)	34 (18.3)	1.000	0.555
3–4	72 (76.6)	57 (82.6)		169 (81.3)	152 (81.7)		
ICH, *n* (%)	48 (51.1)	40 (58.0)	0.428	52 (25.0)	63 (33.9)	0.059	**< 0.001**
EVD, *n* (%)	31 (33.0)	15 (21.7)	0.158	92 (44.2)	83 (44.6)	1.000	**< 0.001**
Current smoker, *n* (%)	49 (52.1)	40 (58.0)	0.525	102 (49.0)	89 (47.9)	0.840	0.194
History of hypertension, *n* (%)	30 (31.9)	31 (44.9)	0.103	85 (40.9)	84 (45.2)	0.416	0.257
Endovascular treatment, *n* (%)	1 (1.1)	0 (0.0)	1.000	43 (20.7)	146 (78.5)	**< 0.001**	**< 0.001**
Multiple aneurysms treated, *n* (%)	19 (20.2)	13 (18.8)	1.000	21 (10.1)	7 (3.8)	**0.018**	**< 0.001**

*aSAH *aneurysmal subarachnoid hemorrhage, *IQR *Interquartile range, *Acom/A1* Anterior communicating artery/Anterior cerebral artery (1st segment), *ICA* Internal carotid artery (includes cavernous, communicating, and paraopthalmic segments), *MCA* Middle cerebral artery, *VBA/PCA* Vertebrobasilar arteries/Posterior cerebral artery, *PICA/AICA/SCA* Posterior inferior cerebellar artery/Anterior inferior cerebellar artery/Superior cerebellar artery, *WFNS *World Federation of Neurological Surgeons, *ICH* Intracerebral hemorrhage, *EVD* External ventricular drainage

## Case fatality by treatment era

Among all 825 aSAHs (including sudden deaths and conservatively treated patients), we observed a nonsignificant increase (from 34.2 to 37.4%; unadjusted RR = 1.09 (95% CI 0.91–1.31)) in the 1-year CFR between surgical and endovascular eras (Table 2). Conversely, we found a significant decrease (from 14.2 to 8.2%; unadjusted RR = 0.58 (95% CI 0.35–0.95)) in 1-year CFRs among the 557 patients who underwent aneurysm repair (Table 2). This decrease occurred similarly in the 163 MCA patients (from 14.9% to 8.7%; unadjusted RR = 0.58 (95% CI 0.24–1.45)) and the 394 non-MCA aSAH patients (from 13.9 to 8.1%; unadjusted RR = 0.58 (95% CI 0.32–1.05)) (Table 2). Based on our univariate analysis, increasing age, poor clinical condition on admission, thick bleeding, presence of ICH, need of EVD, increasing aneurysm size, and nonsmoking were associated with increased 1-year CFR among operatively treated aSAH patients (Supplementary Table 3). Since aneurysm size was the only variable that also changed differently between the aneurysm groups over the treatment transition (decreased in the MCA group but did not change in the non-MCA group), our final multivariable model included the variables of age, sex, aneurysm location, and aneurysm size. According to this adjusted multivariable model, the CFR decreases remained similar between MCA (adjusted RR = 0.66 (95% CI 0.16–1.60)) and non-MCA aneurysms (adjusted RR = 0.66 (95% CI 0.37–1.19)) (*p* = 0.963 for trend difference). All findings also occurred similarly for 30-day CFRs (Table 2).

**Table 2:** Changes in 30-day and 1-year aSAH CFRs by treatment era

	Surgical Era (2012–2014)	Endovascular Era (2015–2017)	Univariable model		Multivariable model^a^	
	deaths/cases (%)	deaths/cases (%)	Risk ratio (95% CIs)	*p*-value	Adjusted risk ratio (95% CIs)	*p*-value
Operated MCA cases						
30-day CFR	4/94 (4.3)	2/69 (2.9)	0.68 (0.13–3.63)	0.653	0.82 (0.15–4.51)	0.824
1-year CFR	14/94 (14.9)	6/69 (8.7)	0.58 (0.24–1.45)	0.245	0.66 (0.16–1.60)	0.353
Operated non-MCA cases						
30-day CFR	13/208 (6.3)	7/186 (3.8)	0.60 (0.25–1.48)	0.268	0.69 (0.27–1.76)	0.432
1-year CFR	29/208 (13.9)	15/186 (8.1)	0.58 (0.32–1.05)	0.070	0.66 (0.37–1.19)	0.168
Operated MCA and non-MCA cases						
30-day CFR	17/302 (5.6)	9/255 (3.5)	0.63 (0.28–1.38)	0.248	0.69 (0.30–1.57)	0.372
1-year CFR	43/302 (14.2)	21/255 (8.2)	0.58 (0.35–0.95)	**0.030**	0.66 (0.40–1.07)	0.091
All aSAH cases						
30-day CFR	117/427 (27.4)	132/398 (33.2)	1.21 (0.98–1.49)	0.072	1.16 (0.95–1.42)^b^	0.139
1-year CFR	146/427 (34.2)	149/398 (37.4)	1.09 (0.91–1.31)	0.331	1.05 (0.89–1.25)^b^	0.540

^a^Adjusted for age, sex, aneurysm group (MCA vs. non-MCA), and aneurysm size

^b^Adjusted for age and sex

*aSAH*  Aneurysmal subarachnoid hemorrhage, *CFR* Case fatality rate, *MCA* Middle cerebral artery

The likelihood ratio test showed interaction *p*-values of 0.897 and 0.963 for trend differences in the adjusted 30-day and 1-year CFRs between MCA and non-MCA cases, respectively

## Discussion

In this six-year follow-up study of a high-volume neurosurgical center, we found similar CFR trends between aSAH patients with ruptured non-MCA and MCA aneurysms, even though the first-line treatment modality of non-MCA aneurysms underwent a fast paradigm shift from surgery to endovascular operations. Given that the proportion of operatively treated patients and prevalence of the two most important outcome predictors (poor clinical condition on admission and thick bleeding detected via brain CT-scan) did not change in either group over the treatment shift, our findings provide real-world evidence that the independent effect of endovascular treatment modalities on decreasing aSAH CFRs may be small. In other words, it is conceivable that improvements in other outcome-related factors such as increased availability of brain CT scans [31], shortened treatment delays [23], improved neurocritical care [22], decreased population-wide prevalence of lifestyle-related risk factors [5, 18] and preventive treatments (also smoking cessation) among patients with high-risk unruptured intracranial aneurysms [15, 16] may have contributed more substantially to the decreasing CFRs of aSAH than the gradual implementation of endovascular treatment.

Several studies have attributed the reduction in aSAH CFRs to the gradually increasing utilization of endovascular treatment [20, 26, 27, 34], but previous research on outcomes during a fast treatment paradigm shift has remained limited, with conflicting findings [1, 12, 28]. Our results are in line with previous single-center studies from the UK undergoing a similar fast implementation of endovascular treatment in 2001–2003 (from 39 to 68%) [12] and 2004–2006 (from 12 to 69%) [1] following the publication of the first ISAT results [25]. These studies reported a nonsignificant tendency toward improved 6-month functional outcomes among aSAH patients in the endovascular era. However, they had relatively small sample sizes (177 and 139 aSAH patients), did not identify nonhospitalized aSAH patients, or conduct subgroup analyses by aneurysm location. With similar limitations, a previous US study based on the National Inpatient Sample reported a significant decrease (from 27 to 24%) in in-hospital aSAH CFRs between 2000–2002 and 2004–2006, while the rate of endovascular treatment increased from 8 to 43% [28]. 

Regardless of the limitations of the ISAT, including the underrepresentation of MCA aneurysms (14% of all aneurysms), posterior circulation aneurysms (3% of all aneurysms), and poor-grade aSAHs patients (5% of all patients), its results are commonly extrapolated to consider all aSAH patients in a real-world clinical practice [32]. This has led to an avid implementation of endovascular treatment to all aneurysms in many centers [32]. However, the modern treatment of aSAH in most high-volume institutions involves a case-specific approach, as surgery may be better suited for some aneurysms while endovascular treatment is optimal for others. For example, ruptured MCA aneurysms often have a broad neck, present with an ICH requiring evacuation, and still have high recanalization rates after endovascular treatment (even 20% at 1-year follow-up), which makes them generally suitable for surgical repair [13, 17, 35, 36]. Conversely, posterior circulation aneurysms are commonly characterized by their complex exposure, proximity to vital structures, and rarity of ICH [14], so transitioning to endovascular treatment has likely improved outcomes for this patient group (as found in the Barrow Ruptured Aneurysm Trial [24]). This is also supported by our findings where 1-year CFRs tended to decrease more in posterior circulation aneurysms (54%; from 24 to 11%) than in MCA (42%; from 15 to 9%) or other anterior circulation aneurysms (42%; from 12 to 7%) over time (Table 2) (Supplementary Table 4). For other locations, namely the anterior cerebral artery (ACA) and the internal carotid artery (ICA), both interventions have remained as suitable repairing modalities of RIAs [4]. Indeed, 37 out of 149 (25%) ACA/ICA aneurysms were still treated surgically in our neurosurgical center during the endovascular era, most commonly due to the presence of ICH requiring surgical evacuation (14 out of 37), patients’ young age (6 out of 37), challenging aneurysm projection (4 out of 37), and wide base of the aneurysm (4 out of 37). Therefore, more studies are needed to investigate CFR differences based on aneurysm location and characteristics, rather than focusing on patients with so-called “equipoise” aneurysms (i.e., cases suitable for both treatment modalities according to specialists), which do not represent the real-world aSAH patients.

### Limitations

This study has limitations. First, as we focused solely on CFRs and did not assess functional outcomes or major complications (rebleeding [8] and delayed cerebral ischemia [8]), possible divergent trends in these factors were missed. However, the study did not aim at focusing on these more ambiguous outcome measures that primarily require a prospective data collection for reliable assessment. Second, flow diverters were not widely used for aSAH at HUH until 2017, and the endovascular treatment consisted mainly of coiling (93%) and intrasaccular devices (6%) during our study period (Supplementary Table 5). However, this likely only has a negligible impact on the generalizability of our findings, as flow diversion is rarely used as a first-line treatment for aSAH [11]. Third, since this study was based on aSAH patients treated in a single high-volume academic center in Finland, the results may not be generalizable to centers with different case-volumes, or to regions with different population and healthcare structures. However, conducting a similar study in multiple ISAT-like centers presents challenges, as most of these centers have made the treatment transition gradually without monitoring sudden deaths and conservatively treated patients. Lastly, it can be speculated that the limited endovascular experience in our center may have undermined the CFR improvements among non-MCA cases. However, similar to many high-volume centers worldwide [6], endovascular treatments of aneurysms have already been performed at HUH neurosurgery since the 1990s.

## Conclusion

Although we found decreasing aSAH CFRs during a fast treatment shift from surgical to endovascular operations of RIAs, the decreases were similar between the aneurysm group that experienced a dramatic increase in endovascular treatment (non-MCA cases) and the group which was almost entirely treated surgically in both treatment eras (MCA cases). Therefore, the reduction in aSAH CFRs may be more attributable to improvements in other outcome-related factors than to the gradual implementation of endovascular treatment.

## Supplementary Information

Below is the link to the electronic supplementary material.

Supplementary File 1 (DOCX 24.2 KB )

## Data Availability

The datasets analyzed in the study are not publicly available and cannot be shared for public use due to the data privacy restrictions of our study approvals. Nevertheless, eligible researchers may request permission to access the datasets from the local institutional review board of HUH, and through the Finnish Social and Health Data Permit Authority (https://findata.fi/en/).

## Abbreviations

ACA: Anterior cerebral artery
aSAH: Aneurysmal subarachnoid hemorrhage
CDR: Cause of Death Register
CFR: Case fatality rate
CI: Confidence interval
CRHC: Care Register for Health Care
EVD: External ventricular drainage
HUH: Helsinki University Hospital
MCA: Middle cerebral artery
ICA: Internal carotid artery
ICH: Intracerebral hemorrhage
ISAT: International Subarachnoid Aneurysm Trial
PPV: Positive predictive value
RIA: Ruptured intracranial aneurysm
RR: Risk ratio
WFNS: World Federation of Neurological Surgeons
