# Supplementary material for: Has a fast treatment transition from surgical to endovascular operations improved the survival of aneurysmal subarachnoid hemorrhage?
Source: Acta Neurochir (Wien). 2025 Feb 4;167(1):34. doi: 10.1007/s00701-025-06447-1 (PMC11794335; doi:10.1007/s00701-025-06447-1)
Supplement: Supplementary file 1 — (DOCX 24.2 KB ) [file 701_2025_6447_MOESM1_ESM.docx]

**Supplementary Table 1.** Definitions of patient characteristics, scales, imaging findings, and their methods of collection.

| **Variable** | **Definition and collection method** |
| --- | --- |
| Aneurysm location | Data on aneurysm location was based on the last CTA, MRA, or DSA imaging before aneurysm repair. Cerebrovascular fellows at our center screened the images and localized all aneurysms (Acom/A1, ICA, MCA, pericallosal, VBA/PCA, and PICA/AICA/SCA). Images were retrieved via the nationwide PACS. |
| Aneurysm size | Data on aneurysm size was based on the last CTA, MRA, or DSA imaging before aneurysm repair. Cerebrovascular fellows at our center screened the images and measured maximum dome diameter in millimeters for all aneurysms. Images were retrieved via the nationwide PACS. |
| Treatment modality | Data on the treatment modality (surgical vs endovascular) and endovascular techniques were collected using surgical or radiological reports. Data was collected by the first author. |
| Treatment of multiple aneurysms | Data on the treatment of multiple aneurysms was collected using surgical or radiological reports. Data was collected by the first author. |
| WFNS grade  (aSAH severity) | Poor-grade aSAH was defined as WFNS grade 4–5 (GCS score <13) and good-grade aSAH was defined as WFNS grade 1–3 (GCS score ≥13). WFNS grading was based on the GCS score (evaluated by a medical doctor) on admission to neurosurgery or on the last reliable assessment before intubation and sedation. If the GCS score was absent from health records, the first author calculated GCS scores based on neurological examination findings. |
| Modified Fisher grade (Thickness of bleeding) | Thick bleeding was defined as modified Fisher grade 3–4 (≥4mm thick diffuse or local layer on the first CT-scan) and thin/absent bleeding as modified Fisher grade 0–2 (<4mm thin layer on the first CT-scan). Scaling was performed by the first author based on the first available noncontrast CT-scan after admission to any emergency room. Images were retrieved via the nationwide PACS. |
| ICH | Data on the presence of ICH (defined as any intraparenchymal bleed on a CT-scan regardless of location or size) was based on the first available noncontrast CT-scan after admission to any emergency room. The first author screened the images for the presence of ICH, and with inconclusive cases, the decision was based on radiology reports. Images were retrieved via the nationwide PACS. |
| EVD | Data on the placement of an EVD was collected through surgical reports. Data was collected by the first author. |
| History of hypertension | History of hypertension was defined as a mention of diagnosed hypertension or use of antihypertensive medication before aSAH on any health record. History of hypertension is one of the systematically asked questions upon hospital admission, hospital discharge, and at the postoperative doctor’s appointment. Thus, it is likely that most patients without a mention of diagnosed hypertension or use of antihypertensive medication truly did not have a history of hypertension. Data was collected by the first author. |
| Current smoking | Current smoking was defined as a mention of active smoking within 6 months before aSAH on any health record, or mention of active smoking in any neurosurgical record after aSAH. History of smoking is one of the systematically asked questions upon hospital admission, hospital discharge, and at the postoperative doctor’s appointment. Thus, it is likely that most patients without a mention of active smoking were truly nonsmokers. Data was collected by the first author. |

Abbreviations: aSAH = Aneurysmal subarachnoid hemorrhage; CTA = Computed tomography angiography; MRA = Magnetic resonance angiography; DSA = Digital subtraction angiography; Acom/A1 = Anterior communicating artery/Anterior cerebral artery (1^st^ segment); ICA = Internal carotid artery (includes cavernous, communicating, and paraopthalmic segments); MCA = Middle cerebral artery; VBA/PCA = Vertebrobasilar arteries/Posterior cerebral artery; PICA/AICA/SCA = Posterior inferior cerebellar artery/Anterior inferior cerebellar artery/Superior cerebellar artery; PACS = Picture Archiving and Communication System; WFNS = World Federation of Neurological Surgeons; GCS = Glasgow Coma Scale; ICH = Intracerebral hemorrhage; EVD = External ventricular drain.

**Supplementary Table 2.** Distribution of treatment strategies in treated aSAH cases by treatment era.

|  | **Surgical Era (2012–2014)** | **Endovascular Era (2015–2017)** |
| --- | --- | --- |
| Aneurysm location | Endovascular treatment/all cases (%) | Endovascular treatment/all cases (%) |
| ACom/A1 | 15/93 (16.1) | 72/98 (73.5) |
| Pericallosal | 1/13 (7.7) | 4/8 (50.0) |
| MCA | 1/94 (1.1) | 0/69 (0.0) |
| ICA | 15/65 (23.1) | 35/43 (81.4) |
| VBA/PCA | 8/24 (36.0) | 23/23 (100.0) |
| PICA/AICA/SCA | 4/13 (30.8) | 12/14 (85.7) |

Abbreviations: aSAH = Aneurysmal subarachnoid hemorrhage; Acom/A1 = Anterior communicating artery/Anterior cerebral artery (1^st^ segment); ICA = Internal carotid artery (includes cavernous, communicating, and paraopthalmic segments); MCA = Middle cerebral artery; VBA/PCA = Vertebrobasilar arteries/Posterior cerebral artery; PICA/AICA/SCA = Posterior inferior cerebellar artery/Anterior inferior cerebellar artery/Superior cerebellar artery.

**Supplementary Table 3.** Risk factors for 30-day and 1-year CFR among operatively treated aSAH patients according to univariate analysis.

|  | **30-day CFR** | | **1-year CFR** | |
| --- | --- | --- | --- | --- |
|  | Risk ratio (95% CI) | P-value | Risk ratio (95% CI) | P-value |
| Age increase per year | 1.02 (0.98–1.07) | 0.289 | 1.05 (1.03–1.08) | <0.001 |
| Male sex | 0.58 (0.24–1.42) | 0.233 | 0.64 (0.38–1.10) | 0.109 |
| WFNS grade (4-5) | 14.46 (4.39–47.60) | <0.001 | 7.40 (4.13–13.27) | <0.001 |
| Modified Fisher grade  (3-4) | N/A (predicts CFR perfectly) | N/A | 4.83 (1.55–15.13) | 0.007 |
| ICH | 0.92 (0.42–2.03) | 0.843 | 1.98 (1.25–3.13) | 0.004 |
| EVD | 8.36 (2.92–23.96) | <0.001 | 5.43 (3.08–9.59) | <0.001 |
| Current smoking | 0.73 (0.34–1.55) | 0.408 | 0.59 (0.37–0.96) | 0.033 |
| History of hypertension | 1.04 (0.49–2.23) | 0.914 | 1.04 (0.65–1.65) | 0.877 |
| Aneurysm location |  |  |  |  |
| Acom/A1 | 1.00 (0.34–2.91) | 0.994 | 0.81 (0.45–1.47) | 0.488 |
| Pericallosal | N/A (no fatal events) | N/A | N/A (no fatal events) | N/A |
| MCA | 1.00 (reference) |  | 1.00 (reference) |  |
| ICA | 2.01 (0.72–5.64) | 0.184 | 0.91 (0.46–1.78) | 0.773 |
| VBA/PCA | 1.16 (0.24–5.55) | 0.856 | 1.56 (0.76–3.20) | 0.224 |
| PICA/AICA/SCA | 3.02 (0.80–11.36) | 0.102 | 1.21 (0.45–3.26) | 0.710 |
| MCA vs non-MCA | 1.38 (0.56–3.37) | 0.481 | 0.91 (0.55–1.50) | 0.710 |
| Aneurysm size per 1mm increase | 1.07 (1.00–1.14) | 0.058 | 1.08 (1.05–1.11) | <0.001 |
| Endovascular treatment | 1.02 (0.46–2.25) | 0.956 | 0.76 (0.45–1.27) | 0.288 |
| Treatment of multiple aneurysms | 1.51 (0.54–4.23) | 0.437 | 1.72 (0.95–3.11) | 0.073 |

Abbreviations: aSAH = aneurysmal subarachnoid hemorrhage; CFR = Case fatality rate; WFNS = World Federation of Neurological Surgeons; ICH = Intracerebral hemorrhage; EVD = External ventricular drainage; Acom/A1 = Anterior communicating artery/Anterior cerebral artery (1^st^ segment); ICA = Internal carotid artery (includes cavernous, communicating, and paraopthalmic segments); MCA = Middle cerebral artery; VBA/PCA = Vertebrobasilar arteries/Posterior cerebral artery; PICA/AICA/SCA = Posterior inferior cerebellar artery/Anterior inferior cerebellar artery/Superior cerebellar artery.

**Supplementary Table 4.** The 30-day and 1-year CFRs in operatively treated non-MCA aSAH cases by aneurysm circulation and treatment era.

|  | **Surgical Era (2012–2014)** | **Endovascular Era (2015–2017)** | **Univariable model** | | **Multivariable model^a^** | |
| --- | --- | --- | --- | --- | --- | --- |
|  | deaths/cases (%) | deaths/cases  (%) | Risk ratio  (95% CIs) | p-value | Risk ratio  (95% CIs) | p-value |
| **Anterior circulation** |  |  |  |  |  |  |
| 30-day | 10/171 (5.9) | 5/149 (3.4) | 0.57 (0.20–1.64) | 0.301 | 0.70 (0.23–2.12) | 0.531 |
| 1-year | 20/171 (11.7) | 11/149 (7.4) | 0.63 (0.31–1.28) | 0.200 | 0.77 (0.38–1.56) | 0.460 |
| **Posterior circulation** |  |  |  |  |  |  |
| 30-day | 3/37 (8.1) | 2/37 (5.4) | 0.67 (0.12–3.81) | 0.648 | 1.02 (0.17–6.15) | 0.984 |
| 1-year | 9/37 (24.3) | 4/37 (10.8) | 0.44 (0.15–1.33) | 0.146 | 0.47 (0.16–1.43) | 0.185 |

Abbreviations: aSAH = Aneurysmal subarachnoid hemorrhage; CFR = Case fatality rate; MCA = Middle cerebral artery

^a^Adjusted for age, sex, and aneurysm size.

The likelihood ratio test showed interaction p-values of 0.990 and 0.461 for trend differences in the adjusted 30-day and 1-year CFRs between anterior and posterior circulation cases, respectively.

**Supplementary Table 5.** Distribution of the utilized endovascular technique by treatment era.

|  | **Surgical Era (2012–2014)** | **Endovascular Era (2015–2017)** |
| --- | --- | --- |
| Endovascular technique, n/N (%) |  |  |
| Coiling with or without stenting | 44/44 (100.0) | 133/146 (91.1) |
| Intrasaccular device | 0/44 (0.0) | 12/146 (8.2) |
| Parent artery occlusion | 0/44 (0.0) | 1/146 (0.7) |
